# How Online Quality Ratings Influence Patients’ Choice of Medical Providers: Controlled Experimental Survey Study

**DOI:** 10.2196/jmir.8986

**Published:** 2018-03-26

**Authors:** Niam Yaraghi, Weiguang Wang, Guodong (Gordon) Gao, Ritu Agarwal

**Affiliations:** ^1^ Department of Operations and Information Management University of Connecticut Stamford, CT United States; ^2^ Center for Technology Innovation The Brookings Institution Washington, DC United States; ^3^ Department of Decision, Operations and Information Technologies Robert H Smith School of Business University of Maryland at College Park College Park, MD United States

**Keywords:** quality of health care, health care evaluation mechanisms

## Abstract

**Background:**

In recent years, the information environment for patients to learn about physician quality is being rapidly changed by Web-based ratings from both commercial and government efforts. However, little is known about how various types of Web-based ratings affect individuals’ choice of physicians.

**Objective:**

The objective of this research was to measure the relative importance of Web-based quality ratings from governmental and commercial agencies on individuals’ choice of primary care physicians.

**Methods:**

In a choice-based conjoint experiment conducted on a sample of 1000 Amazon Mechanical Turk users in October 2016, individuals were asked to choose their preferred primary care physician from pairs of physicians with different ratings in clinical and nonclinical aspects of care provided by governmental and commercial agencies.

**Results:**

The relative log odds of choosing a physician increases by 1.31 (95% CI 1.26-1.37; *P*<.001) and 1.32 (95% CI 1.27-1.39; *P*<.001) units when the government clinical ratings and commercial nonclinical ratings move from 2 to 4 stars, respectively. The relative log odds of choosing a physician increases by 1.12 (95% CI 1.07-1.18; *P*<.001) units when the commercial clinical ratings move from 2 to 4 stars. The relative log odds of selecting a physician with 4 stars in nonclinical ratings provided by the government is 1.03 (95% CI 0.98-1.09; *P*<.001) units higher than a physician with 2 stars in this rating. The log odds of selecting a physician with 4 stars in nonclinical government ratings relative to a physician with 2 stars is 0.23 (95% CI 0.13-0.33; *P*<.001) units higher for females compared with males. Similar star increase in nonclinical commercial ratings increases the relative log odds of selecting the physician by female respondents by 0.15 (95% CI 0.04-0.26; *P*=.006) units.

**Conclusions:**

Individuals perceive nonclinical ratings provided by commercial websites as important as clinical ratings provided by government websites when choosing a primary care physician. There are significant gender differences in how the ratings are used. More research is needed on whether patients are making the best use of different types of ratings, as well as the optimal allocation of resources in improving physician ratings from the government’s perspective.

## Introduction

To improve quality, foster competition, promote transparency, and help patients make informed decisions, it is critical for patients to have access to reliable information and make cognizant choices about their medical providers [1,2]. In recent years, a concerted effort in the United States has been put in place to develop and publicly report quality measures of medical providers [3].

The Centers for Medicare and Medicaid Services (CMS) is the most prominent governmental agency in the United States that collects, aggregates, and reports quality measures of different aspects of medical care. Through initiatives such as Hospital Compare [4], CMS reports quality data on both clinical and nonclinical aspects of medical services offered by different providers. Surgical complications, infections, readmission, and death rates are examples of metrics that measure the clinical aspects of medical care. Surveys of patients’ experiences, such as the Hospital Consumer Assessment of Healthcare Providers and Systems, capture metrics that measure nonclinical aspects of care. In parallel with CMS, private and commercial agencies such as Vitals [5], RateMDs [6], and ProPublica [7] also collect and report quality metrics on both clinical and nonclinical aspects of care. Recent research shows that although the ratings provided by commercial agencies may be inconsistent with each other [8], they are more comprehensive and cover a broader range of domains than what is included in ratings reported by CMS [9,10].

Ratings of health care providers are growing in importance and popularity [11-18], affecting both the revenue and the reputation of medical providers [19-22]. For example, when CMS released its quality metrics of nursing homes to the public, the market share of 1-star facilities decreased by 8%, whereas the market share of 5-star facilities increased by more than 6% [23]. Similar effects have also been documented for hospitals [24]. Although nonclinical ratings provided by commercial agencies are correlated with the conventional measures of patient experience as reported by governmental agencies [25,26], the relationship between patient reviews and medical outcomes is not clear. Some studies find that patient satisfaction reported as nonclinical ratings is not associated with clinical outcomes [27-32], whereas others report a strong association between these two types of ratings [33,34]. For a review of literature on the association between the social media reviews and the clinical quality outcomes, see Verhoef et al [35].

Despite the significant differences between the types (clinical and nonclinical) and the sources (governmental and commercial agencies) of ratings, variations in their relative significance for patient choice of medical providers are not known. The purpose of this research was to fill this gap by uncovering the relative importance of these ratings in the decision-making processes of different groups of patients.

## Methods

### Data Source

We used a primary dataset consisting of responses of 1000 individuals who were each paid 50 cents to participate in an online experiment through Amazon Mechanical Turk (AMT) in October 2016. These individuals were all master users of AMT and live in the United States. According to AMT, a user achieves a master distinction by consistently completing requests with a high degree of accuracy. Masters must continue to pass AMT’s statistical monitoring to maintain their status [36].

Table 1 provides a comparison of demographics between the sample in this study and the US population. In contrast to the US population, our sample consisted of less affluent, but more educated, younger adults. Although, when compared with the US population, our sample of AMT users consisted of younger and more technologically savvy individuals, we relied on this sample to conduct our analysis for the following reasons. First, given the question posed in this research, the sample did not need to be representative of the US population and, instead, only had to represent individuals who used information resources available on the Internet. As this study compared the importance of two information resources that are exclusively Web-based, its sample also had to include the individuals who could use resources on the Web. Second, prior research shows that despite limitations, data that are gathered from “AMT samples are at least as reliable as those obtained via traditional methods. Overall, AMT can be used to obtain high-quality data inexpensively and rapidly” [37].

### Study Design

To determine how ratings on different attributes affect individuals’ evaluations of medical providers, we designed an experiment and conducted a choice-based conjoint analysis [38] as a rigorous method of eliciting preferences [39]. We describe the method below.

The combination of 2 categories (clinical and nonclinical) and 2 sources (governmental and commercial agencies) resulted in 4 different types of ratings: clinical ratings provided by a governmental agency, nonclinical ratings provided by a governmental agency, clinical ratings provided by a commercial agency, and nonclinical ratings provided by a commercial agency. In this research, we use “governmental agency” and “public agency” interchangeably. We assigned a high or low value to each type of rating, and thereby created 16 profiles of hypothetical physicians. In a 1-to-4-star rating system, to induce appropriate variation, we used 2 stars to indicate low ratings and 4 stars to indicate high ratings. Each profile represented a physician with different ratings on the 4 categories. These profiles were *balanced*, which means that each of the 2 levels (2 and 4 stars) in each of the 4 types of ratings appeared the same number of times in physician profiles. Using these 16 profiles, we then created 8 pairs of physicians such that the 4 types of ratings in each pair were *orthogonal* [40]. This ensured that any pair of levels from different rating types appeared the same number of times in the design. We used % *mktex* [41] macro in SAS software (version 9.4) to create the balanced and orthogonal design. Table 2 shows the 16 profiles in 8 pairs.

In a Web-based interface, we first provided respondents with a brief tutorial on different sources and types of ratings. Specifically, we described the public agency as “the department of Health and Human Services, which is a branch of the federal government” and the commercial agency as “websites such as Yelp, RateMDs, Healthgrades, Vitals, Zocdoc, and DoctorScorecard.”

**Table 1:** Characteristics of 949 respondents and the US population.

Variable and class		Sample, n (%)		Percentage of US population^a^ (%)	*H*_0_: *P*_USA_−*P*_Sample_=0^b^ (*z* value)
**Education**					
	Advanced degree	114 (12.0)		10.38	−1.65^c^
	Bachelor’s degree	381 (40.2)		18.88	−16.74^d^
	Associate’s degree	100 (10.5)		5.28	−7.25^d^
	Some college, no degree	231 (24.3)		19.42	−3.83^d^
	Trade or technical school	30 (3.2)		4.08	1.43
	Graduated high school	90 (9.5)		29.63	13.59^d^
	Less than high school	3 (0.3)		12.33	11.25^d^
**Income, US $**					
	150,000 or more	30 (3.2)		13.57	9.36^d^
	125,000-149,999	25 (2.6)		5.42	3.8^d^
	100,000-124,999	70 (7.4)		8.71	1.45
	75,000-99,999	123 (13.0)		12.26	−0.66
	50,000-74,999	227 (23.9)		16.96	−5.71^d^
	35,000-49,999	170 (17.9)		12.92	−4.58^d^
	25,000-34,999	136 (14.3)		9.39	−5.22^d^
	Less than 25,000	168 (17.7)		20.77	2.33^e^
**Race**					
	Asian	56 (5.9)		5.70	−0.27
	Black	66 (7.0)		13.30	5.76^d^
	Hawaiian	1 (0.1)		0.20	0.62
	Hispanic	53 (6.0)		17.8	9.84^d^
	Indian	16 (1.7)		1.30	−1.06
	White	757 (79.8)		76.90	−2.1^e^
**Marital status**					
	Divorced	74 (7.8)		9.80	2.07^e^
	Married/Domestic partner	471(49.6)		51.87	1.38
	Separated	8 (0.8)		2.09	2.69^d^
	Single/Never married	390 (41.1)		32.25	−5.83^d^
	Widowed	6 (0.6)		5.72	6.75^d^
**Gender**					
	Female	548 (57.7)		50.80	−4.28^d^
	Male	401 (42.3)		49.20	4.28^d^
**Age**					
	Younger than 65 years	924 (97.4)		87.00	−9.49^d^
	65 years and older	25 (2.6)		13.00	9.49^d^

^a^Authors’ analysis of characteristics of experiment participants. Demographics of US population are calculated based on the data provided by the US Census Bureau.

^b^The null hypothesis that the percentage in sample is equal to that of the US population.

^c^*P*<.10.

^d^*P*<.01.

^e^*P*<.05.

**Table 2:** Physician profiles used in choice-based conjoint experiment. “gGvernment” indicates that a public agency provides the ratings, and “Commercial” indicates that a private organization provides the ratings. In the Web-based interface, the hypothetical physician profiles in each pair were shown side-by-side and respondents were asked to choose the physician they prefer. The sequence of the pairs and the attributes in each profile were generated randomly to ensure that the order of the presentation of rank of the attributes did not influence the respondent’s choice. The values of 2 or 4 in the table, respectively, indicate a “2” or “4” star rating in the physician profiles provided to respondents in the Web-based experiment.

Pair number^a^	Government rating		Commercial rating	
	Clinical	Nonclinical	Clinical	Nonclinical
One	2; 4	4; 2	2; 4	4; 2
Two	2; 4	4; 2	4; 2	4; 2
Three	2; 4	2; 2	2; 2	4; 2
Four	4; 2	2; 4	2; 4	4; 2
Five	4; 2	2; 4	4; 2	4; 2
Six	4; 2	4; 2	2; 4	4; 2
Seven	2; 4	2; 4	4; 2	4; 2
Eight	2; 4	2; 4	2; 4	2; 4

**Figure 1:**
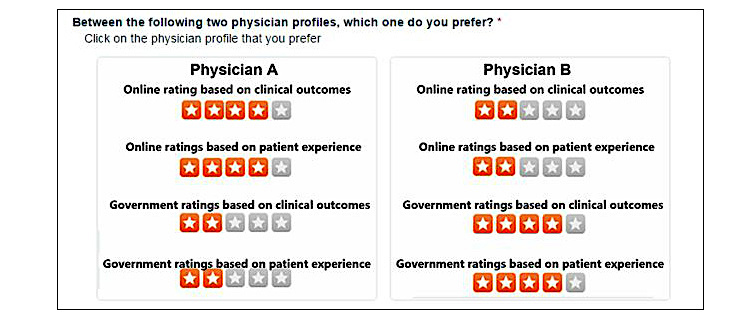
Screenshot of the choice-based conjoint experiment.

We also distinguished clinical and nonclinical ratings and explained to the survey respondents that clinical ratings by the public agency were determined “based on official statistics on how often physicians provide care that research shows leads to the best results for patients” and nonclinical ratings by the public agency were determined based on “a national survey that asks patients about their experiences with staff, nurses, and doctors during a recent visit to the doctor.” Similarly, we explained that clinical ratings provided by the commercial agency were determined by “the patient online reviews about how patients evaluate the medical expertise of the doctor” and nonclinical ratings provided by the commercial agency were created based on “patient online reviews about their experiences with staff, nurses, and doctors during a recent visit to the doctor.” To assess if respondents correctly distinguished the differences between the types and the sources of ratings, at the end of the survey, we asked them to describe each type of the ratings in their own words. Our examination of their responses confirmed that all respondents had fully understood different ratings.

We then presented the 8 pairs of hypothetical profiles of physicians in a random sequence and asked respondents to choose the physician they prefer in each pair. A screenshot of 1 of the 8 comparison pairs is presented in Figure 1, which corresponds to the choices in pair Seven as shown in Table 1. To simulate a realistic decision-making scenario, we asked the respondents to imagine that they have moved to a new town and have to choose a new primary care physician based solely on the 4 types of ratings provided to them. This approach ensured that the choice of the respondents in our experiment was only driven by the ratings and was not confounded by any other factor outside of our model, such as insurance coverage, location, or race of the physician [42,43].

Once respondents finished the evaluation of physicians in the 8 pairs, we asked them a series of questions designed to evaluate their health status, medical literacy, trust in Web-based reviews, and trust in government as 4 composite indexes. We conducted factor analysis to operationalize these 4 constructs using validated items that we derived from prior literature in information systems [44,45] and medicine [46,47]. Details on the items, composite indexes, and factor analysis are provided in Multimedia Appendix 1.

One potential concern with the study design was that respondents may not complete the choice task thoughtfully. To detect and filter the responses that were provided hastily and without careful attention, we included 2 trap questions in the experiment.

The first trap question was the choice of physicians in the eighth pair (shown in Table 2), one of which was superior on all of the 4 types of ratings and clearly dominated the pair. A respondent’s choice of an inferior physician indicated lack of attention to the experiment. The second trap question asked, “How happy will you be if you receive a letter from Internal Revenue Service that says you should pay a large amount of taxes to the government?” We assumed that a respondent did not pay attention to the question if she chose “extremely happy” or “happy” as a response to this question.

### Statistical Analysis

Our research design fit the multinomial logit model with clustered error terms [48,49]. Following the suggestions of Kuhfeld [50], we used the PHREG [51] procedure in SAS software for the estimation. In this model, the dependent variable was binary and indicated the choice that a respondent made from a pair of hypothetical physician profiles. The 4 types of ratings in each profile constituted our main independent variables. In the multinomial logit model used in this study, the probability that a respondent chose a specific physician in a pair was a function of the attributes of that specific physicians as well as the attributes of the other physician in the pair. The PHREG [51] procedure in SAS not only allowed us to account for the conditional dependency of choices for the alternatives in a pair but also adjusted for the correlation between the 8 choices made by the same respondent. Using this model, we could examine the relative importance of the 4 types of ratings. We further explored whether patient attributes, such as age, gender, and income, moderated the impact of the ratings. To statistically compare the effects of different regression coefficients, we implemented the tests provided by Paternoster et al [52].

## Results

On the basis of the answers to the 2 trap questions, we excluded 51 observations from our initial sample of 1000 responses. We retained the remaining 949 responses for further analysis (Table 1). We present the estimation results of our multinomial logit model in Table 3.

As shown in the last (full model) column of Table 3, the relative log odds of choosing a physician increased by 1.31 (95% CI 1.26-1.37; *P*<.001) and 1.32 (95% CI 1.27-1.39; *P*<.001) units when the government clinical ratings and commercial nonclinical ratings moved from 2 to 4 stars, respectively. The importance of these 2 types of ratings was statistically equivalent (*P*=.49). By comparison, the relative log odds of choosing a physician increased by a modest 1.12 (95% CI 1.07-1.18; *P*<.001) units when the commercial clinical ratings moved from 2 to 4 stars. The relative log odds of selecting a physician with 4 stars in nonclinical ratings provided by the government was 1.03 (95% CI 0.98-1.09; *P*<.001) units higher than a physician with 2 stars in this rating. The difference between the effects of government nonclinical ratings and commercial clinical ratings on patients’ choice of a primary care physician were statically significant (*P*=.04). The difference between the effects of clinical ratings provided by government and those provided by a commercial agency was statistically significant (*P*<.001). Likewise, the difference between the government clinical ratings and the government nonclinical ratings was also statistically significant (*P*<.001).

One standard deviation improvement in a patient’s health status increased the relative log odds of choosing a physician with 4 stars in commercial nonclinical ratings by 0.18 (95% CI 0.13-0.24; *P*<.001) units and decreased the relative log odds of choosing a physician with 4 stars in government clinical ratings by 0.14 (95% CI 0.08-0.19; *P*<.001) units.

Medical literacy had no statistically significant effect on how patients evaluated different types of ratings. As the level of trust in overall Web-based ratings increased, the importance of nonclinical ratings provided by a commercial agency also increased. One standard deviation increase in a patient’s trust in Web-based reviews increased the relative log odds of choosing a physician with 4 stars in nonclinical commercial ratings by 0.07 (95% CI 0.02-0.13; *P*=.05) units. Unsurprisingly, as the patients’ level of trust in the government increased, the importance of clinical ratings provided by government increased, whereas the importance of nonclinical ratings provided by a commercial agency decreased.

One standard deviation increase in a patient’s trust in government increased the relative log odds of choosing a physician with 4 stars in government clinical ratings by 0.20 (95% CI 0.15-0.25; *P*<.001) units and decreased the relative log odds of choosing a physician with 4 stars in commercial nonclinical ratings by −0.15 (95% CI 0.10-0.21; *P*<.001) units. These trends remained consistent even when we included more variables in our model. We also examined how patients’ demographic characteristics of gender, race, income, education, marital status, and age affected the importance of each of the 4 ratings in their evaluation of primary care physicians. Table 4 presents the results. The log odds of selecting a physician with 4 stars in nonclinical government ratings relative to a physician with 2 stars was 0.23 (95% CI 0.13-0.33; *P*<.001) units higher for females compared with males. Similar star increase in nonclinical commercial ratings increased the relative log odds of selecting the physician by female patients by an additional 0.15 (95% CI 0.04-0.25; *P*=.006) units, compared with males.

**Table 3:** The relative importance of different types and sources of ratings on patients’ choice. GC: clinical ratings provided by a public agency (government). GNC: nonclinical ratings provided by a public agency (government). YC: clinical ratings provided by a commercial agency (commercial). YNC: nonclinical ratings provided by a commercial agency (commercial).

Parameter^a^	Parameter estimate (95% CI)					
	Basic model	Health status	Medical literacy	Trust in online reviews	Trust in government	Full model
GC	1.29^b^ (1.24 to 1.34)	1.29^b^ (1.24 to 1.35)	1.29^b^ (1.24 to 1.34)	1.29^b^ (1.24 to 1.34)	1.30^b^ (1.25 to 1.35)	1.31^b^ (1.26 to 1.36)
GNC	1.00^b^ (0.95 to 1.05)	1.01^b^ (0.96 to 1.06)	1.00^b^ (0.95 to 1.05)	1.00^b^ (0.95 to 1.05)	1.01^b^ (0.96 to 1.06)	1.03^b^ (0.98 to 1.08)
YC	1.09^b^ (1.04 to 1.14)	1.11^b^ (1.06 to 1.16)	1.10^b^ (1.04 to 1.14)	1.10^b^ (1.04 to 1.14)	1.11^b^ (1.07 to 1.16)	1.12^b^ (1.07 to 1.18)
YNC	1.29^b^ (1.24 to 1.34)	1.31^b^ (1.25 to 1.36)	1.29^b^ (1.24 to 1.34)	1.29^b^ (1.24 to 1.35)	1.30^b^ (1.25 to 1.35)	1.32^b^ (1.27 to 1.37)
Health status × GC		−0.13^c^ (−0.18 to −0.08)				−0.13^c^ (−0.19 to −0.08)
Health status × GNC		0.09^c^ (0.04 to 0.14)				0.10^c^ (0.05 to 0.15)
Health status × YC		0.05 (0 to 0.10)				0.05 (0 to 0.10)
Health status × YNC		0.17^b^ (0.12 to 0.22)				0.18^b^ (0.13 to 0.23)
Medical literacy × GC			0 (−0.06 to 0.04)			0 (−0.06 to 0.04)
Medical literacy × GNC			0 (−0.05 to 0.04)			−0.01 (−0.05 to 0.04)
Medical literacy × YC			0.03 (−0.01 to 0.08)			0.03 (−0.01 to 0.08)
Medical literacy × YNC			−0.01 (−0.07 to 0.03)			−0.01 (−0.07 to 0.03)
Online trust × GC				0.06^d^ (0.01 to 0.11)		0.06 (0.01 to 0.11)
Online trust × GNC				0.02 (−0.02 to 0.07)		0.02 (−0.02 to 0.07)
Online trust × YC				−0.05 (−0.10 to 0)		−0.05 (−0.10 to −0.01)
Online trust × YNC				0.07^d^ (0.02 to 0.12)		0.07^d^ (0.02 to 0.12)
Trust in government × GC					0.19^b^ (0.14 to 0.24)	0.20^b^ (0.14 to 0.25)
Trust in government × GNC					0.01 (−0.04 to 0.05)	0.01 (−0.04 to 0.05)
Trust in government × YC					0.03 (−0.02 to 0.08)	0.02 (−0.02 to 0.08)
Trust in government × YNC					−0.14^b^ (−0.20 to −0.09)	−0.15^b^ (−0.20 to −0.10)

^a^Authors’ analysis of revealed choices in the choice-based conjoint analysis. Health status, medical literacy, online trust, and trust in government are composite indexes, centered around mean 0 with standard deviation of 1; 95% CI are reported in parentheses.

^b^*P*<.001.

^c^*P*<.01.

^d^*P*<.05.

**Table 4:** Interaction of ratings and patient characteristics. GC: clinical ratings provided by a public agency (government). GNC: nonclinical ratings provided by a public agency (government). YC: for clinical ratings provided by a commercial agency (commercial). YNC: the nonclinical ratings provided by a commercial agency (commercial).

Parameter	Parameter estimate (SE)						
	Female	White	High income	High education	Married	Age	Full model
GC	1.31^a^ (0.05)	1.24^a^ (0.08)	1.25^a^ (0.05)	1.18^a^ (0.05)	1.26^a^ (0.05)	1.15^a^ (0.12)	1.03^a^ (0.14)
GNC	0.87^a^ (0.05)	1.16^a^ (0.08)	0.98^a^ (0.04)	1.03^a^ (0.05)	0.97^a^ (0.04)	1.22^a^ (0.11)	1.25^a^ (0.14)
YC	1.15^a^ (0.05)	1.15^a^ (0.08)	1.08^a^ (0.05)	1.11^a^ (0.05)	1.07^a^ (0.04)	1.32^a^ (0.11)	1.42^a^ (0.14)
YNC	1.19^a^ (0.05)	1.30^a^ (0.08)	1.24^a^ (0.05)	1.29^a^ (0.05)	1.18^a^ (0.05)	1.21^a^ (0.12)	1.13^a^ (0.14)
GC × Female	−0.03 (0.05)						−0.04 (0.05)
GNC × Female	0.23^a^ (0.05)						0.23^a^ (0.05)
YC × Female	−0.09 (0.05)						−0.10 (0.05)
YNC × Female	0.18^b^ (0.05)						0.15^b^ (0.05)
GC × White		0.05 (0.09)					0.03 (0.10)
GNC × White		−0.19^c^ (0.09)					−0.17 (0.09)
YC × White		−0.08 (0.09)					−0.05 (0.09)
YNC × White		−0.01 (0.09)					−0.04 (0.10)
GC × Income			0.074 (0.07)				0.01 (0.08)
GNC × Income			0.02 (0.07)				0.02 (0.07)
YC × Income			0.01 (0.07)				0 (0.07)
YNC × Income			0.10 (0.07)				0.03 (0.08)
GC × Education				0.21^b^ (0.07)			0.21^b^ (0.07)
GNC × Education				−0.06 (0.07)			−0.07 (0.07)
YC × Education				−0.04 (0.07)			−0.04 (0.07)
YNC × Education				0 (0.07)			−0.01 (0.07)
GC × Married					0.05 (0.07)		0.03 (0.08)
GNC × Married					0.05 (0.07)		0.046 (0.07)
YC × Married					0.04 (0.07)		0.09 (0.08)
YNC × Married					0.22^b^ (0.07)		0.18^c^ (0.08)
GC × Age						0.003 (0.003)	0.01 (0.01)
GNC × Age						−0.006^c^ (0.002)	−0.006^c^ (0.003)
YC × Age						−0.006^c^ (0.002)	−0.006^c^ (0.003)
YNC × Age						0.002 (0.003)	0 (0.003)

^a^*P*<.001.

^b^*P*<.01.

^c^*P*<.05.

## Discussion

### Principal Findings

To the best of our knowledge, this was the first research that, using a conjoint analysis, uncovered how individuals used Web-based ratings to compare and choose medical providers. We found that the clinical ratings provided by the government and the nonclinical ratings provided by a commercial agency were significantly more important for patient choice than nonclinical ratings provided by the government or clinical ratings provided by commercial agencies. We also found some differences in the importance of ratings based on the sociodemographic and health characteristics of respondents. Healthier patients paid more attention to nonclinical ratings, especially those from a commercial agency. On the other hand, for healthier patients, the importance of clinical ratings, notably those that are provided by the government, was lower. We found that female patients gave more importance to nonclinical ratings provided by both public and commercial agencies, compared with males. In comparison with other races, white respondents paid less attention to the nonclinical ratings provided by government. There was no other difference between racial groups in the importance of different types of ratings in the physician choice decision. Income did not play a role in the way respondents used the ratings in their decision. As patients get older, nonclinical ratings provided by the government and the clinical ratings provided by a commercial agency became even less important in how they evaluated medical providers.

A particular strength of this study was that we utilized a carefully controlled experimental design to observe the revealed preferences of participants rather than merely asking them to state them in response to a questioner, which could otherwise be subject to attribution or social desirability biases. Revealed preferences elicited in this experiment provided a more natural context, even when presented in hypothetical settings, and gave us greater confidence that the effects we observed within the sample were driven by the conjoint attributes rather than other unobserved factors.

### Limitations

One limitation of our study was that we rated the attributes of the physicians by either 2 or 4 stars, whereas in reality, the ratings usually have 5 levels, between 1 and 5 stars. We limited the ratings to only 2 levels to reduce the number of possible combinations. If we considered 5 levels for each rating, the number of possible physician profiles would have surged from 16 to 625. Respondents could not reasonably compare these many physician profiles with each other. A second limitation of this study was that, in comparison with the US general population, its sample was drawn from younger, more educated, and less affluent individuals. Although samples from AMT have been shown to respond similarly to representative samples of the US population [37], the results from the study must be interpreted in light of the characteristics of the sample. Third, this study only focused on American respondents, and therefore, findings may not generalize to individuals outside of the United States. This was due to the fact that constructs such as medical literacy, health status, and trust in government significantly vary across individuals from different countries. Moreover, the presence of commercial websites and the availability of alternative government websites also vary across countries, which represents a further limitation on generalizability. Finally, in our study, we did not ask respondents whether they were familiar with the sources of information they were being asked to evaluate, primarily because our major focus was on the source (ie, government vs commercial) rather than a specific website. Future experiments could also ask respondents about their familiarity with the sources of information that they are asked to evaluate in the experiment.

### Future Research

There are 3 potential areas for further research. The first is to examine how familiar individuals are with the sources of information provided by governmental and commercial agencies. Although most individuals are now fairly familiar with the commercial rating websites, knowledge about the other sources of information provided through governmental websites may be limited. It would be useful to quantify the level of awareness of such information as a precursor to designing appropriate policies to inform the public. The second is to replicate this study on an international sample to investigate how individuals outside of the United States rely on different sources and types of information for choosing their primary care physicians. Finally, the relative importance of Web-based ratings in comparison with other factors such as insurance coverage, recommendations of family and friends, and proximity to patients’ residence is still unclear and could be investigated in future research.

### Policy Recommendations

The findings of this research have implications for policy makers and medical providers. Although the government has expended substantial resources on clinical quality ratings, our study indicates a need to also acknowledge the importance of nonclinical measures. This is consistent with the recent CMS efforts and policy recommendations [53] to tie reimbursements to patient satisfaction. To the extent that nonclinical ratings appear to be more important for healthier patients, it clearly underscores the important role played by the “experience” of interacting with a physician for individuals whose visits to the doctor are likely to be preventive rather than curative. Primary care providers can consider ways in which the patient’s experience can be improved, such as reduced waiting time and more empathetic interactions, which will eventually be reflected in the nonclinical ratings they receive. The results of this study could also encourage a public relations campaign to increase public awareness of the reviews that are government maintained and are more clinically based. Our result on gender differences in the relative salience of nonclinical ratings further revealed the importance of improving the patient experience for providers who are focused on women’s health services.

With respect to patients’ age, we found that older patients and those who trusted government more paid more attention to government-provided ratings. This is corroborated by prior literature, which documents that citizens who trust government more are also more satisfied with government websites [54]. We therefore recommend that CMS create website content and user experiences that are tailored for Medicare beneficiaries and older patients as they rely on government-provided information more than the younger patients. Our results also indicated that commercial websites can be more successful in attracting younger individuals. If CMS intends to expand its audience, it should consider information dissemination strategies that appeal to patients in this segment.

Given the recent apprehensions expressed about the quality and representativeness of ratings provided by commercial websites [55], it is a matter of some concern that patients gave equal importance to commercial ratings of nonclinical aspects of care much as they did to government ratings of clinical aspects of care. This is likely a result of the richness of the information that patients believe they can receive from other patients who have engaged in interactions with the medical provider. It might also be driven by other factors such as the first mover advantage of commercial organizations as they have been active in rating a wide variety of services earlier than other governmental agencies. To that end, our findings suggest that patients have developed a preference for commercial websites for experience-based ratings of medical providers, that is, ratings that primarily capture information about the patient’s experience with the medical provider. Thus, government agencies that offer similar ratings should pay careful attention to improve the usability of the information while concurrently addressing any perceptual obstacles that may prevent consumers from using these ratings.

### Conclusions

Our research shows that patients pay equal attention to both clinical and nonclinical ratings when choosing a primary care physician. To obtain information about clinical ratings, they rely more on government sources, whereas for information on nonclinical ratings, they rely more on commercial sources. Both public and private agencies expend significant resources to design metrics, collect data, calculate ratings, and report them to the public. These resources are limited and should be optimally allocated to the type of ratings that consumers appreciate and will use the most. The findings of this research highlight the importance of efforts from government agencies such as CMS to improve its reporting of nonclinical ratings. Given the importance of nonclinical ratings in patients’ decision making, we recommend that medical providers pay close attention to their nonclinical ratings on commercial websites as they represent a consequential source of customer feedback for improving the patient experience. Ultimately, the overarching objective of all rating sources must be focused on protecting patients from incorrect or misleading data, while simultaneously educating them on how best to interpret and make best use of the information presented.

## Appendix

Multimedia Appendix 1 How online quality ratings influence patients’ choice of medical providers: a controlled experimental survey study appendix (online-only material).

## Abbreviations

AMT: Amazon Mechanical Turk
CMS: Centers for Medicare and Medicaid Services
