# Supplementary material for: How Online Quality Ratings Influence Patients’ Choice of Medical Providers: Controlled Experimental Survey Study
Source: J Med Internet Res. 2018 Mar 26;20(3):e99. doi: 10.2196/jmir.8986 (PMC5891665; doi:10.2196/jmir.8986)
Supplement: Multimedia Appendix 1 [file jmir_v20i3e99_app1.pdf]

Factor Analysis

To create the composite indexes, we use the following question items presented in Table A1. Descriptive statistics of responses to these questions are presented in Table A2. The loadings of the items on four factors are shown in Table A3. The reliably and validity measures of the composite indexes are presented in Table A4.

Importance of Ratings for Different Groups of Patients

The interaction between four types of ratings and patient characteristics (gender, race, income, education, marital status and age) are presented in Table A5. All the variables expect age are binary.

“Female“ is equal to one if gender is female. “White” is equal to one if race is white, “high income“ is equal to one if income is higher than $50,000 per year. “High education” is equal to one is education level is equal or higher than Bachelor’s level. “Married” is equal to one if the individual is married or in a domestic partnership.

Table A1: Question items used in the survey to measure composite indexes

Composite	Code	Item	Source
Health Status	HS1	How frequently do you schedule doctor appointments for yourself?	Ware and Sherbourne (1992)
	HS2	How many different prescription medications are you taking for chronic or long-term health problems?	
	HS3	Select any of the following health conditions that you have ever been diagnosed with by a doctor or other health professional? (a set of 11 medical conditions is provided)	
	HS4	In general, how would you rate your overall health?	
Medical Literacy	ML1	How confident are you filling out medical forms by yourself?	Chew et al. (2004), Wallston et al. (2014)
	ML2	How often do you have someone help you read medical material (such as hospital notes, prescription information or doctor orders)?	
	ML3	How often do you have problems learning about your medical condition because of difficulty understanding written information?	
Trust in Online Reviews	OR1	When making a major purchase such as an appliance, a smartphone, or even a car, how important are on-line reviews in your decision making?	Sherry Bonelli (2016)
	OR2	How often do you read online customer reviews to determine whether a local business is a good business?	
	OR3	How often do you trust online customer reviews as much as	

		personal recommendations?	
	OR4	How many online reviews do you need to read before you feel that you can trust that business?	
Trust in Government	GT1	To what extent do you agree with the following statements:<br>"I believe that the government, in general, would act in my best interest"	McKnight et al. (2002)
	GT2	"If I required help, the government would do its best to help me"	
	GT3	"The government, in general, is interested in my well-being, not just their own"	
	GT4	“The government is truthful in dealings with me”	
	GT5	“The government would keep commitments”	
	GT6	“The government is sincere and genuine”	

Table A2: Descriptive Statistics

Variable	Description	Min	Max	Mean	S.D.
Health Status (HS)					
HS1	Frequency of doctor appointments<br>*Less than once a year=5, More than once a month=1*	1	5	3.86	1.04
HS2	Number of medications<br>*None=5, More than 6=1*	1	5	4.42	0.87
HS3	Self-evaluation of overall health<br>*Excellent=5, Poor=1*	1	5	3.46	0.91
HS4	Number of chronic conditions<br>*None=5, More than 4=1*	1	5	4.45	0.86
Medical Literacy (ML)					
ML1	Confidence in filling medical forms by yourself<br>*Extremely=5, Not at all=1*	1	5	4.22	0.87
ML2	Help from others to understand medical material<br>*Never=5, Always=1*	1	5	4.3	1.03
ML3	Difficulty to understand written medical info<br>*Never=5, Always=1*	1	5	4.4	0.87
Trust in online ratings (OR)					
OR1	Importance of online ratings ion purchase decision<br>Extremely important=5, Not important at all=1	1	5	3.9	0.87
OR2	Read online reviewers to determine quality of a business<br>*Always=5, Never=1*	1	5	3.67	0.92
OR3	Trust online reviews as personal recommendations<br>*Always=5, Never=1*	1	5	3.36	0.84
OR4	Number of reviews needed to trust a business<br>*"1 or 2"=5, "More than 20"=5*	1	5	2.89	0.97
Trust in Government (GT)					
GT1	Government acts in my best interest<br>*Strongly agree=5, Strongly disagree=1*	1	5	2.89	1.03
GT2	Government does its best to help me<br>*Strongly agree=5, Strongly disagree=1*	1	5	2.75	1.01
GT3	Government is interested in my well-being<br>*Strongly agree=5, Strongly disagree=1*	1	5	2.65	1.06
GT4	Government is truthful<br>*Strongly agree=5, Strongly disagree=1*	1	5	2.76	0.99
GT5	Government keeps its commitments<br>*Strongly agree=5, Strongly disagree=1*	1	5	2.83	1.04
GT6	Government is genuine and sincere<br>*Strongly agree=5, Strongly disagree=1*	1	5	2.47	0.98

Table A3: Rotated factor matrix (loading) of factors

Variable	Factor 1	Factor 2	Factor 3	Factor 4
**HS1**	-3	**64**	-37	-11
**HS2**	0	**77**	-23	-17
**HS3**	7	**64**	-14	1
**HS4**	7	**72**	-21	-18
**ML1**	-7	13	7	**66**
**ML2**	-16	7	-15	**81**
**ML3**	-15	17	-19	**81**
**OR1**	-11	26	**76**	8
**OR2**	-5	32	**76**	10
**OR3**	-4	28	**74**	-5
**OR4**	0	-12	-29	-1
**GT1**	**88**	0	7	7
**GT2**	**88**	2	6	6
**GT3**	**89**	3	4	5
**GT4**	**87**	-1	0	8
**GT5**	**85**	0	-3	7
**GT6**	**88**	-1	1	3

Table A4: Properties of measurement model

Construct and indicators	Standardized Loading	Indicator Reliability	Error Variance	Composite reliability	Cronbach Alpha	Discriminate Validity
Health Status				0.736424	0.723929	0.418825
HS1	0.60961	0.3716	0.6284			
HS2	0.79797	0.6368	0.3632			
HS3	0.48120	0.2316	0.7684			
HS4	0.65978	0.4353	0.5647			
Medical Literacy				0.721385	0.698171	0.479433
ML1	0.42622	0.1817	0.8183			
ML2	0.76958	0.5923	0.4077			
ML3	0.81505	0.6643	0.3357			
Trust in Online Reviews				0.769359	0.768557	0.5268
OR1	0.74795	0.5594	0.4406			
OR2	0.73760	0.5441	0.4559			
OR3	0.69055	0.4769	0.5231			
Trust Government				0.940459	0.940452	0.724883
GT1	0.83526	0.6977	0.3023			
GT2	0.80525	0.6484	0.3516			
GT3	0.84920	0.7211	0.2789			
GT4	0.86912	0.7554	0.2446			
GT5	0.86680	0.7513	0.2487			
GT6	0.88057	0.7754	0.2246			

References (for online appendix):

1. Ware JE, Sherbourne CD. The MOS 36-Item Short-Form Health Survey (SF-36): I. Conceptual Framework and Item Selection. Med Care. 1992;30(6):473-483.

2. Chew LD, Bradley KA, Boyko EJ. Brief questions to identify patients with inadequate health literacy. Fam Med. 2004;36(8):588-594.

3. Wallston KA, Cawthon C, McNaughton CD, Rothman RL, Osborn CY, Kripalani S. Psychometric properties of the brief health literacy screen in clinical practice. J Gen Intern Med. 2014;29(1):119–126.

4. Sherry Bonelli. Local Consumer Review Survey 2016 | The Impact Of Online Reviews. Bright Local; 2016. https://www.brightlocal.com/learn/local-consumer-review-survey/. Accessed February 17, 2017.

5. McKnight DH, Choudhury V, Kacmar C. Developing and validating trust measures for e-commerce: An integrative typology. Inf Syst Res. 2002;13(3):334–359.
